# Biomarkers of Activation and Inflammation to Track Disparity in Chronological and Physiological Age of People Living With HIV on Combination Antiretroviral Therapy

**DOI:** 10.3389/fimmu.2020.583934

**Published:** 2020-10-09

**Authors:** Michellie Thurman, Samuel Johnson, Arpan Acharya, Suresh Pallikkuth, Mohan Mahesh, Siddappa N. Byrareddy

**Affiliations:** ^1^ Department of Pharmacology and Experimental Neuroscience, University of Nebraska Medical Center, Omaha, NE, United States; ^2^ Department of Microbiology and Immunology, University of Miami School of Medicine, Miami, FL, United States; ^3^ Southwest National Primate Research Institute, Texas Biomedical Research Institute, San Antonio, TX, United States

**Keywords:** HIV, immune aging, inflammatory markers, activation markers, combined antiretroviral therapy

## Abstract

With advancement, prompt use, and increasing accessibility of antiretroviral therapy, people with HIV are living longer and have comparable lifespans to those negative for HIV. However, people living with HIV experience tradeoffs with quality of life often developing age-associated co-morbid conditions such as cancers, cardiovascular diseases, or neurodegeneration due to chronic immune activation and inflammation. This creates a discrepancy in chronological and physiological age, with HIV-infected individuals appearing older than they are, and in some contexts ART-associated toxicity exacerbates this gap. The complexity of the accelerated aging process in the context of HIV-infection highlights the need for greater understanding of biomarkers involved. In this review, we discuss markers identified in different anatomical sites of the body including periphery, brain, and gut, as well as markers related to DNA that may serve as reliable predictors of accelerated aging in HIV infected individuals as it relates to inflammatory state and immune activation.

## Introduction

Aging is a natural process involving decline in physiological integrity and reduced organ function (1). These features can be manifested in the form of chronic degenerative diseases, cancers, inflammation, and cellular senescence. Human Immunodeficiency Virus (HIV) infection shares common factors associated with conditions observed in older, uninfected people. Chronic HIV infection in the setting of anti-retroviral therapy (ART) is commonly associated with inflammaging, low-grade inflammation, a primary contributor to age-related conditions such as increased vulnerability to infections, and development of age associated co-morbid conditions including cancers, cardiovascular diseases (CVD), and neurodegenerative diseases (2, 3). During aging, inflammation is largely driven by cellular senescence, an increase in cellular debris, and microbial translocation (4). These same factors contribute to inflammation in HIV infection. Additionally, HIV viral proteins including gp120, Tat, Vpr, and Nef are able to induce inflammatory signaling independent of these processes in both lymphocytes and myeloid cells (5, 6).

People with HIV (PLWH) are living longer due to widespread use and advancement of combined antiretroviral therapy (cART); the life expectancy of cART-treated HIV-infected individuals was merely 55 years in 1996 and now it mirrors that of the general population (7–9). The advancement of cART dramatically decreases systemic inflammation in HIV-infected people which results in a delay in progression to acquired immunodeficiency syndrome (AIDS) (10). However, while PLWH are reaching older ages, they are not aging normally. Despite cART, PLWH still experience comorbidities, chronic inflammation, and symptoms of premature aging. For example, senescent T cells, characterized by a curbed proliferative state often triggered by stresses such as DNA damage, telomere shortening, or presence of inflammatory cytokines, can accumulate in tissues as they age. PLWH are susceptible to developing other conditions indicative of premature aging, including decline in integrity of neurological function, or HIV-associated neurocognitive disorders (HAND). Even HIV-infected individuals receiving cART are likely to exhibit mild forms of neurological impairment related to HAND (11). Aging is a complex process that involves and affects a multitude of organs creating a need for greater understanding of the biomarkers involved, specifically within the context of HIV. The purpose of this review is to highlight similarities between HIV-associated premature aging and chronological aging by examining biomarkers that define inflammatory state in PLWH, a phenotype for accelerated aging. These biomarkers originate from diverse pathways making it difficult to propose a single mechanism for treatment, however, they offer a holistic understanding of inflammation in PLWH on cART and may present new avenues for therapeutics.

## Immunosenescence and Genetic Aging

### Lymphocytes

**Figure 1:**
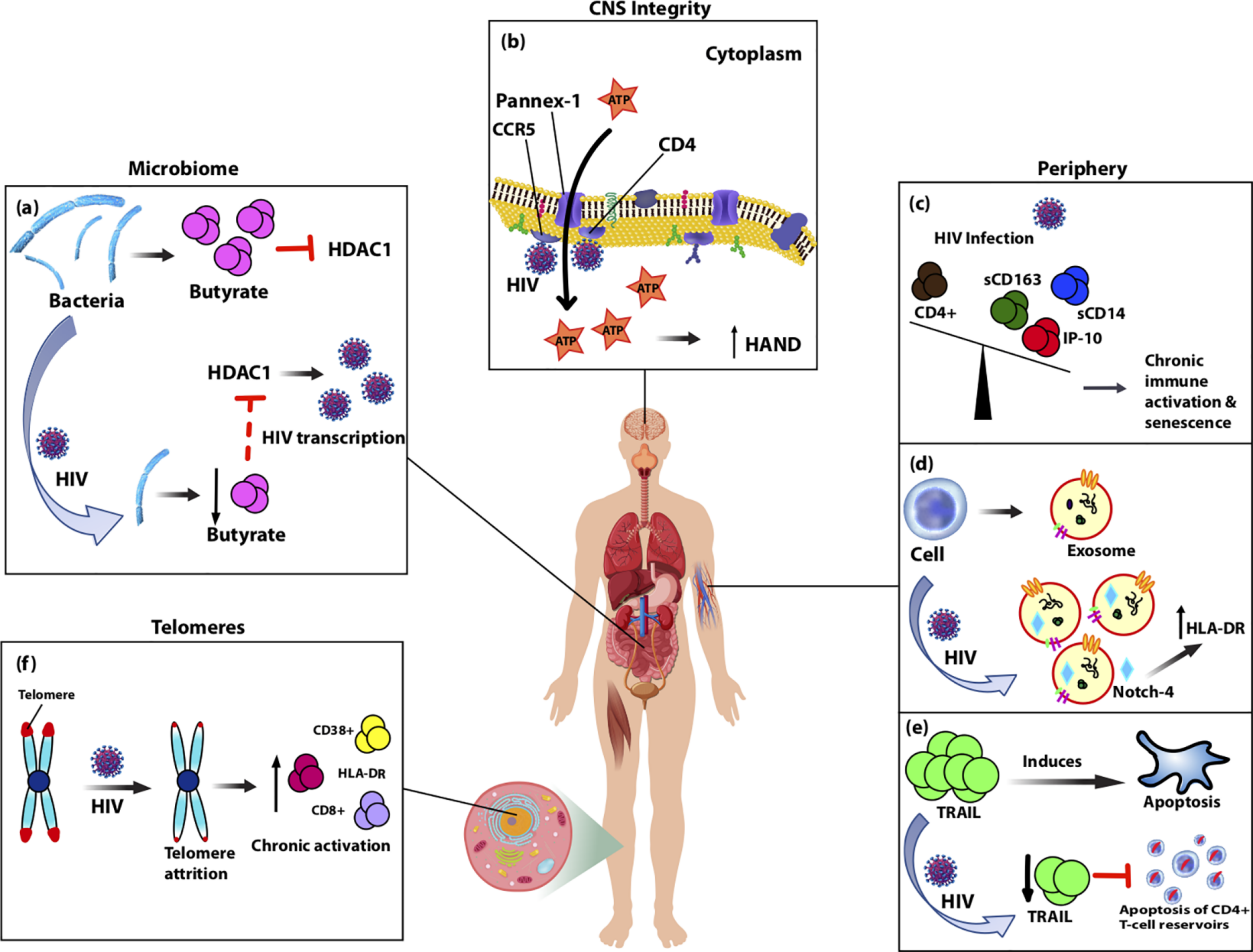
Diagram showing biomarkers isolated from various anatomical sites of the body that are altered by HIV-infection and likely contribute to accelerated aging observed in people living with HIV (PLWH) on cART through chronic immune activation and inflammation. **(A)** Typically, gut-associated bacteria, Firmicutes, produces butyrate which inhibits HDAC1. With normal aging or HIV-infection, Firmicutes is replaced causing reduced production of butyrate and consequently increased expression of HDAC1, which acts to increase HIV transcription. **(B)** Pannex-1 channels, usually closed, open upon binding of HIV to receptors CD4 and co-receptor CCR5, which causes release of ATP, an inflammatory signal. Increased levels of ATP in circulation were correlated with cognitive impairment and thus predictive of CNS compromise. **(C)** During HIV-infection plasma levels of monocyte activation markers sCD163 and sCD14, as well as pro-inflammatory marker IP-10 are elevated and inversely related with CD4+ T-cell depletion. Over-expression of these markers in the periphery leads to accelerated aging of T cells and senescence. **(D)** Upon HIV-infection, secretion of exosomes increases along with oxidative stress markers, and HIV-induced chronic activation alters the contents of exosomes. Notch-4 exosomal levels are elevated and correlated with other activation markers, HLA-DR. **(E)** HIV-infection reduces expression of circulating TRAIL, an apoptosis-inducing protein, which theoretically in turn limits apoptosis of CD4+ T-cell reservoirs allowing for persistent immune activation and inflammation. **(F)** Telomeres undergo attrition after HIV-infection due to reduced T-cell proliferation and this is associated with cellular senescence markers CD8+, HLA-DR, and CD38+.

In PLWH, systemic activation of the immune system and virus-induced cell death drive CD4+ T-cell depletion and CD8+ T-cell expansion. Irrespective of active cART, chronic immune activation and inflammation persist which can lead to non-AIDS related conditions (12). Elevated levels of CD38 and HLA-DR co-expressing CD4+ and CD8+ T cells reflect increased immune activation (13, 14). Comparing parameters for immune activation and aging, similarities include an increase in CD28- T cells, greater levels of activation markers such as CD38/HLA-DR, and a transition of naïve T-cells to memory cells (15). These features contribute to immunosenescence: the reduced and imbalanced effectiveness of the adaptive and innate immune response induced by the aging process (16). In elderly individuals, there is a smaller ratio of CD4+ to CD8+ T cells due to decreased naïve T-cell levels, greater T-cell activation, and higher levels of inflammatory markers (17). During HIV infection, adipose tissue becomes a site for accumulation of latently-infected CD4+ T cells and activation of CD8+ T cells (18). A low CD4/CD8 ratio is associated with chronic inflammation and can also serve as a predictor of decreased subcutaneous fat loss in the cART-treated HIV-infected population; similarly, with chronological aging, there is increased inflammation and body fat redistribution which have been associated with metabolic complications (19). Another marker, IP-10, is associated with aging in HIV. Upon HIV-infection, plasma levels of pro-inflammatory chemokine IP-10 are upregulated and inversely related to CD4+ T cell counts (**Figure 1**) (20). Elevated levels of IP-10 have been shown to suppress T cell and NK cell function, while simultaneously promoting HIV replication (21–23). Furthermore, elevated levels of IP-10 are associated with aging such that after viral suppression with cART, older individuals are more likely to maintain higher IP-10 plasma levels (24). These findings are consistent with the claim that chronic immune activation induces premature aging.

### Telomeres and Senescence

Age-associated decline in function is characterized by telomere shortening, increased level of NK cells, high percentage of activated CD45RO+CD95+ T cells, greater levels of CD8+CD28-CD57+ senescent T cells, and increase in PD-1+ functionally exhaustive cells (25–28). These levels are reliable predictors of aging phenotypes, including immunosenescence. Utilizing animal models can strengthen our understanding of immunosenescence in HIV infection. In a study using Chinese rhesus macaques, SIV infection caused pathogenesis and immunosenescence, but aged-macaques showed accelerated SIV disease progression including more rapid depletion of CD4+ T cells leading to increased levels of lymphocyte exhaustion relative to younger SIV-infected macaques (29). In another study, SIV-infected macaques had higher expression levels of markers for senescence like p53 and p16 in their subcutaneous and visceral adipose tissue (**Table 1**). CD4+ T cells in the human immune system are long-lived and subject to genomic mutations, damage, and replicative pressures, making them susceptible to age-associated irregularities (95). With aging, there is a slow decline in T cell capacity to proliferate which is correlated with telomere attrition, a phenotype of cellular senescence (96). Telomere shortening is an indicator of inflammaging. In managing HIV-infection, ART restores CD4+ T cell counts, though HIV-infected individuals maintain increased levels of immune activation, shown by higher frequencies of CD38+HLA-DR+CD4+ and CD8+ T-cells, and these markers are associated with shorter telomeres and transitively, immunosenescence (**Figure 1**) (97). One mechanism of telomere damage may be attributed to an inefficient DNA repair mechanism that compromises telomere integrity driven by excessive proliferative turnover rate in these long-lived cells. However, HIV-infected individuals on cART exhibit telomeric DNA damage that is not addressed because of an Ataxia-telangiectasia-mutated gene deficiency which leads to age-associated phenotypes such as telomere shortening, CD4+ T cell senescence, and apoptosis (98). Shorter telomeres have also been associated with inflammatory markers including CXCL1, TGF-α, and IL10RA (42). HIV-infection leads to establishment of viral reservoirs in various anatomical sites of the body, primarily in long-lived CD4+ T cells which persist even in the presence of ART. HIV appears to stimulate telomere elongation in latently infected memory CD4+ T cells which are vulnerable to BRAC019, a telomere-targeting drug that causes uncapping and apoptosis (99).

**Table 1:** Effects of natural versus HIV-induced aging on soluble biomarkers and known age-associated diseases*.

Biomarker	Function	System	Effect of HIV infection	Effect of Aging	Age-associated diseases	References
sCD14	Myeloid differentiation marker on monocytes/macrophages; Marker of monocyte activation in HIV	Periphery	Increased	Increased	Cardiovascular Disease	(30–32)
sCD163	Shed by CD163 scavenger receptor specific to monocytes/macrophages; Marker of monocyte activation in HIV	Periphery	Increased	Increased	Cardiovascular Disease, Liver Disease	(33–35)
IP-10	Pro-inflammatory chemokine involved in T-cell generation and trafficking	Periphery	Increased	Increased	Rheumatoid Arthritis	(36, 37)
NDE	Delivers signaling molecules between cells and reflects host cell proteins and nucleic acids	Brain – isolated from blood	Increased	?	Alzheimer’s Disease	(38, 39)
Notch-4	Regulates cell-fate determination, differentiation, proliferation, apoptotic programs	Plasma exosome contents	Increased	Decreased	Alzheimer’s Disease	(40, 41)
SLAMF1	Glycoprotein that delivers downstream signals directing innate and adaptive immune response; Phagocytic properties	Periphery	Increased	Increased	Rheumatoid arthritis, Alzheimer’s Disease	(42–45)
CCL23	Hematopoiesis inhibitor that directs migration of monocytes, macrophages, activated T-lymphocytes	Periphery	Increased	?	Rheumatoid arthritis, human brain injury, Myeloid leukemia	(42, 46–49)
NT3	Supports differentiation of neurons to promote growth	Periphery	Decreased	Decreased	Colorectal Cancer; Neurocognitive decline	(42, 50–52)
TRAIL	Induces apoptosis of tumor/infected cells; promotes CD4+ T-cell death in HIV	Periphery	Decreased	Increased	Alzheimer’s Disease	(42, 53–55)
p53	Tumor suppressor, DNA repair, cell cycle regulation	Periphery	Increased	Increased	Cancer	(56, 57)
p16	Tumor suppressor, cell cycle regulation, neurogenesis regulation	Periphery, CNS	Increased	Increased	Cancer, neurodegeneration	(56–58)
Neopterin	Pteridine metabolite produced primarily during Th1-type	CNS, periphery	Increased	Increased	Chronic inflammation, neurocognitive decline	(59–61)
NFL	Maintains neuronal shape, including axonal diameter	CNS	Increased	Increased	Neurodegeneration (Specifically axon injury)	(62–64)
sCD30	Tumor necrosis factor receptor	CNS, Periphery	Increased	Increased	Cancer, Inflammation, neurodegeneration	(65, 66)
Serum ATP	Energy transfer, signaling, neurotransmitter	From CNS to periphery	Case-dependent increase	Case-dependent increase	Neurodegeneration	(67, 68)
S100B	Cell cycle regulation, neuron survival, inflammatory response	CNS, gut, periphery	Increased	Variable (“Dose dependent”)	Neurodegeneration, cancer, inflammatory bowel disease	(69–71)
Grey/White Matter Volume	Processing, integrating, and coordinating information	CNS	Decreased	Decreased	Neurodegeneration	(72–75)
Ventricle Volume	Storage and transport CSF	CNS	Increased	Increased	Neurodegeneration	(73, 74)
Choline	Cell membrane degradation and inflammation	CNS	Increased	Increased	Neurodegeneration	(76–80)
Myo-Inositol	Gliosis and neuroinflammation	CNS	Increased	Increased	Neurodegeneration	(76, 78, 80, 81)
N-acetyl Aspartate	Neuron viability and integrity	CNS	Decreased	Decreased	Neurodegeneration	(76–81)
Mean Diffusivity	Measure of water flow and loss of myelin	CNS	Increased	Increased	Neurodegeneration	(82–88)
Fractional Anisotropy	Measure of myelin structure and axon integrity	CNS	Decreased	Decreased	Neurodegeneration	(83, 84, 88, 89)
Ratio of Firmicutes to Bacterioidetes	Butyrate production	Micro-biome	Decreased	Decreased	Inflammation, Neurodegeneration,	(90–94)

*Many of these biomarkers may be used to track HIV pathogenesis as well as changes in inflammatory state with chronological aging,

### Epigenetic

Epigenetics refers to mechanisms regulating chromatin structure which has implications in gene expression and genome stability. Epigenetic alterations are indicative of the aging process due to chromatin remodeling and accumulation of DNA mutations. These changes stimulate protective actions such as DNA methylation (100). PLWH exhibited accelerated epigenetic aging of 7.4 years in brain tissue, 5.2 years in periphery, and virally-suppressed PLWH had a 19% increased risk of mortality compared to healthy controls (101, 102). It has been suggested that HIV-mediated chronic inflammation may be an underlying mechanism contributing to epigenetic aging (103–105). Another study suggested inflammation-related SNPs of these pro-inflammatory cytokines are risk factors for accelerated aging in PLWH. While the TNF-α (TNF-α-308G>A) genotype was not associated with epigenetic aging, IL-6 (IL-6-174G>C) C allele carriers and IL-10 (IL-10-592C>A) CC homozygotes showed significantly greater epigenetic aging compared to other genotypes in PLWH (106). Thus, these SNPs may offer unique insights into HIV aging and the accompanying pathophysiological changes.

## Cytokines, Chemokines, and Inflammaging

While cART effectively establishes viral suppression, there is still a heightened pro-inflammatory state in treated individuals, which induces chronic, low-grade systemic inflammation promoting pathophysiological age-associated changes. There are well-documented markers of inflammation associated with HIV-infection such as cytokines including IP-10, interferon-α, IL-6, IL-10, and IL-15 (107). A recent study investigated a large panel of inflammatory soluble biomarkers in plasma to understand the risk of age-associated diseases in PLWH (**Table 1**). Reportedly, compared to healthy controls, PLWH on long-term cART had higher levels of CST5, 4E-BP1, SLAMF1, CCL23, MMP1, ADA, and CD8A and lower levels of NT3, TRAIL, and sCD5 in plasma (42). Additionally, peripheral inflammatory cytokines (d-dimer, IL-6, MCP-1/CCL2, sCD14, and TNF-α) in HIV-infection have been correlated with impaired complex motor performance, though not a HAND-specific biomarker (108). Furthermore, it has been suggested that these proteins are associated with early stages of age-related diseases (**Table 1**). For example, TNF-related-apoptosis-inducing-ligand (TRAIL) selectively induces apoptosis of tumor cells or infected cells. During HIV-infection, TRAIL could promote CD4+ T-cell death, which fuels interest in its use as a unique method of targeting latent HIV-1 CD4+ T-cells (53–55). Decreased levels of TRAIL observed in PLWH on long-term ART may impede eradication of the latent HIV-1 reservoir (**Figure 1**).

## Monocytes and Myeloid Soluble Markers

Monocyte activation is of increasing interest as a mediator of non-AIDS related morbidity and mortality, and circulating biomarkers in plasma including well-characterized sCD14 and sCD163 are used to study monocyte activation (**Table 1**). CD14 is a co-receptor for LPS found predominantly on the monocytic-macrophage cell lineage, which upon stimulation, releases sCD14, a sign of monocyte activation. While LPS can generate this response, other TLR ligands such as flagellin or CpG oligodeoxynucleotides or inflammatory cytokines such as IL-6 could generate the same, suggesting that sCD14 is a nonspecific marker of monocyte activation (30). Similarly, CD163 has restricted expression to monocytes and macrophages (33). LPS acts as a down-regulator of CD163, while IL-6 acts as a potent stimulator, resulting in shedding of sCD163 (109). PLWH have higher plasma levels of sCD14 and sCD163 and these markers are independent predictors of mortality in HIV (**Figure 1**) (110, 111). Measurement of immune activation markers in HIV-infected individuals on long-term cART showed sCD14 and sCD163 levels were persistently elevated in treated HIV-infected patients compared to healthy controls; however, cART showed no effect in lowering sCD14 levels in HIV-infected individuals before or after initiation of treatment, even after 8 years in some cases (42). cART is more effective at limiting plasma sCD163 levels, though relative to uninfected individuals, there is discrepancy on whether levels are comparable or higher (42, 112, 113). Further, CD14+ monocytes were shown to exhibit increased p90RSK activity, which is a reactive oxygen species-sensitive kinase, in the presence of cART which suppresses NRF2-ARE transcriptional activity eliciting a senescent phenotype along with pro-inflammatory responses (114). In another study, HIV cell-associated DNA (in CSF and blood) and sCD163 (in CSF) were significantly correlated with cognitive impairment, particularly executive function, in older adults, but not in young adults (115). Further, it has been suggested that, plasma sCD163 (but not CSF sCD163) is associated with the severity of symptoms related to cognitive impairment (116). Upon HIV-infection, CD14 and CD163 appear to be the primary drivers of accelerated aging of T cells and immunosenescence, which likely occurs due to chronic immune activation, even in the presence of cART.

## CSF and CNS-Specific Markers

HAND is a complex of neurological disorders characterized by severity and development of cognitive impairments such as mental slowness and memory loss, and motor symptoms including poor balance and tremors. HAND is associated with activation of pathways involved in inflammation and aging (117). Despite cART improvements, HAND still affects between 18 to 55% of HIV+ individuals receiving treatment (11, 118–120). Biomarkers would aid in developing therapeutics prior to manifestation of severe pathologies. One specific marker proposed for CNS dysfunction is neopterin (**Table 1**), a pteridine-metabolite produced primarily during Th1-type responses to inflammatory stimuli such as IFN-γ (121). Although serum levels of neopterin are more closely correlated with overall clinical severity, neopterin in the CSF is a marker for innate cell activation specifically in the CNS. Such immune activation can disrupt CNS homeostasis, thereby leading to HAND development. Neopterin has been correlated with neurocognitive decline (59, 60).

Several other CSF biomarkers have been proposed as detailed in **Table 1**. For example, NT3 is a neurotrophic factor that aids in the survival and differentiation of neurons to stimulate growth and has been robustly linked to neurocognitive impairment in PLWH. Lower levels of NT3 were detected in cART-treated HIV-infected individuals (42). Further, higher sCD14 levels have been correlated with impaired neurocognitive function, specifically attention and learning of HIV-infected individuals (31). Recently, sCD30 was shown to co-localize with HIV-1 RNA and DNA in lymphoid tissues. Plasma levels of sCD30 were lower in individuals with cART-suppressed viremia compared to viremic patients. However, sCD30 was elevated in CSF during CNS infection, regardless of peripheral viral load, potentially indicating continued CNS replication and subsequent resultant damage (65). Further, a pannexin-1 (Panx-1)-specific pathway was associated with compromised CNS. Upon HIV binding to CD4 and co-receptors CXCR4 and/or CCR5, Panx-1 channels open to release local intracellular secondary messengers such as ATP and PGE2 from circulating PBMCs (**Figure 1**). Serum ATP levels were positively correlated with HAND severity (67). Finally, a common CSF marker S100B has been correlated with AIDS dementia complex (ADC) severity and predictive of ADC progression (69). However, use of S100B as a biomarker remains controversial (122).

### Neuroimaging

Less invasive methods of assessing CNS changes, including those leading to HAND, have also been studied using advanced neuroimaging techniques such as magnetic resonance imaging (MRI) (**Table 1**). In the absence of other diagnostic markers at the time of HAND diagnosis, an MRI is frequently performed to confirm there are no other CNS pathologies that could be contributing to cognitive changes, including those that occur naturally with aging. Characteristic volumetric changes that occur in HAND include loss of white and grey matter and enlarged ventricle size resulting from the lost parenchymal mass (72–74). Multiple studies have confirmed that HIV and aging are independently associated with grey and white matter loss with the latter more impacted by aging than by HIV status. Such atrophy was associated with lower scores on neuropsychological tests (75, 123). Another MRI application is magnetic resonance spectroscopy. By suppressing the signal of water molecules during analysis, various CNS metabolites can be distinguished by their unique chemical shift peaks. Typical metabolites used specifically to study HAND include choline (cho), myo-inositol (MI), and *N*-acetyl aspartate (NAA). Cho, a marker of cell membrane degradation and inflammation, is increased in HIV+ individuals (76, 77), particularly among HAND patients (78, 79). This trend is especially pronounced in the basal ganglia (77, 80). These changes were even more pronounced in older individuals (78). MI, a marker of gliosis and neuroinflammation, is also increased in HAND, specifically in the basal ganglia (78, 80). The opposite trend has been noted for NAA, a marker of neuron viability and integrity, with greatest decreases in the basal ganglia and white matter (76–79, 81, 93).

### Microbiome

During HIV pathogenesis, chronic inflammation is driven partially by continued low-level viral replication; however, increased microbe and microbial byproduct translocation across the damaged intestinal epithelial barrier increase inflammation, especially lipopolysaccharide (LPS) (124). LPS is able to induce pro-inflammatory cytokine secretion in myeloid cells after binding to the CD14/TLR4/MD2 receptor complex. Because LPS can drive much of the pathogenesis, serum levels can be monitored to better understand the source of inflammation during HIV pathogenesis. Additional markers associated with gut damage like intestinal fatty acid-binding protein (I-FABP) and other proteins upregulated in the presences of LPS, including LPS-binding protein and sCD14, can also be used as markers of microbiome changes (110, 125, 126). Similarly, (1→3)-β-D-Glucan (βDG), a major component of fungal cell walls can also be used as a marker of microbial translocation (127, 128). As humans age, there is progressive replacement of butyrate-producing Firmicutes with Bacteroidetes, particularly Prevotella (90, 91) and the same pattern has been observed in HIV-infected individuals (92–94). Butyrate is a short-chain fatty acid metabolite of dietary fiber fermentation used as the primary energy source for colonocytes and the promotion of Treg differentiation, thereby protecting the intestinal epithelial barrier from damage (129, 130). Butyrate may also as an HDAC1 inhibitor (HDI) serving as a latency-reversing agent for T cell viral reservoirs (**Figure 1**) (131, 132). Butyrate produced in the gut can also alter CNS health increasing neurogenesis protecting against neurodegeneration (133, 134).

Studies documenting changes that occur after pro-biotic supplementation further highlight the role of the microbiome in inflammation, HIV pathogenesis, and associated aging. In one study, patients who received probiotic cocktails had a reduction in CD4+ T cell activation markers, including HLA-DR and CD38. High sensitivity C-reactive protein (hsCRP) and LBP were also reduced to levels comparable to uninfected controls (135). In another study, HIV-infected patients given *Lactobacillus casei Shirota* supplements had increased T cell counts. This was accompanied by reduced mRNA levels of TGFβ, IL-1β, IL-10, and IL-12, as well as an increase in IL-23 (136). Furthermore, a recent study demonstrated that a probiotic cocktail increased serum serotonin levels and decreased tryptophan, as well as decreased surface markers CD38 and HLA-DR on CD4+ T-cells and mRNA expression of IDO and IFN-γ in PBMCs (137). Finally, another study correlated indolamine-2,3-dioxygenase (IDO) expression in the gut-associated lymphoid tissue (GALT) with levels of neopterin in CSF, both of which were significantly decreased with probiotic supplementation (138). These examples demonstrate that changes to the microbiome during HIV pathogenesis may not only serve as biomarkers of inflammation, but may ultimately be a source of the inflammaging processes and a target for therapeutic interventions.

## Exosomes

Exosomes may be another potential biomarker for HIV pathogenesis. Exosomes are ~30–150 nm extracellular vesicles released from various cell types into plasma, urine, CSF, and inflammatory tissues. The content of exosome cargoes can be changed depending on pathological state or health of the secreting cell, particularly by immune activation (139). Exosomes of different origins can be obtained from the periphery. A recent study investigated proteins associated with neuronal damage in plasma neuron-derived exosomes (NDE) within the context of HIV-infection to differentiate age-associated from HIV-associated neurocognitive decline. NDE counts were positively correlated with age only in HIV-infected subjects, not in their seronegative controls (38). An earlier investigation reported premature cellular senescence caused an increase in the secretion of exosomes (140). Further, another study characterized plasma exosomes isolated from virally suppressed PLWH on cART with respect to immune responses and oxidative stress. They observed higher levels of exosomes in PLWH on cART compared to seronegative controls and found a positive correlation with oxidative stress markers such as CD14, CRP, HLA-A, and HLA-B and a potentially novel one, Notch4 among the exosome contents (141). Notch-4 is involved in regulating cell-fate determination and influencing the differentiation, proliferation, and apoptotic programs of myeloid and dendritic cells (40). Remarkably, exosomal Notch-4 presence was correlated with HLA-DR and decreased CD4/CD8 ratio, which are reliable predictors of immune activation, thus identifying Notch-4 as a promising new biomarker of immune activation in HIV-infection (**Figure 1**) (141). Further, exosomes originating from HIV-1 infected T cells are reportedly involved in several processes of infection such as production of pro-inflammatory cytokines in macrophages, CD4+ T-cell apoptosis, and neurotoxicity (142–144). These studies collectively suggest that exosomes may serve as a novel avenue for examining mechanisms of premature aging and senescence during HIV-infection (**Table 1**).

## Conclusion

Complications observed in PLWH today are CVD, liver disease, neurological decline, non-AIDS related cancers, and metabolic disorders. These are features of normal aging occurring at an earlier chronological age in the HIV-infected population due to persistent chronic immune activation and inflammation. While cART has successfully extended the lives of PLWH, some of these premature aging comorbidities are exacerbated by cART. Therefore, it is important to continue investigating biomarkers of the premature aging process with the aim of developing new treatment avenues and improving quality of life for PLWH. The markers reported throughout this review arise from divergent pathways, and many of them are associated with specific pathology, such as CNS-specific markers. Clinicians should be aware of these diverse mechanisms and potential sources of inflammation, at which point interventions may be necessary. Regardless of current clinical implications, understanding biomarkers of inflammation and aging that are associated with HIV will allow for development of better therapeutics. Additionally, non-human primate model studies offer an ideal avenue for experimentally investigating these biomarkers as they relate to inflammation and thus the pathology of accelerated aging in HIV.

## Author Contributions

MT and SJ convinced the ideas and prepared draft. AA, SP, and MM edited and contributed to the draft. SB convinced the ideas, design, and edited the draft. All authors contributed to the article and approved the submitted version.

## Funding

This work was supported in part by NIH grants, R01AI129745, P30MH062261, R01DA052845, and R01AI113883.

## Conflict of Interest

The authors declare that the research was conducted in the absence of any commercial or financial relationships that could be construed as a potential conflict of interest.

The handling editor declared a past co-authorship with one of the authors SB.
